# Unusual recurrent nevi: To use or not to use reflectance confocal microscopy

**DOI:** 10.1016/j.jdcr.2025.12.020

**Published:** 2026-01-09

**Authors:** Yaelle Shaked, Michael Lee, Ata S. Moshiri, Amanda Levine

**Affiliations:** a Ronald O. Perelman Department of Dermatology, New York University Grossman School of Medicine, New York, New York; b Icahn School of Medicine at Mount Sinai, New York, New York

**Keywords:** atypical nevus, dermoscopy, malignant melanoma, prior biopsy, recurrent nevus, reflectance confocal microscopy, unusual recurrent nevi

## Case report

### Clinical presentation

**Fig 1:**
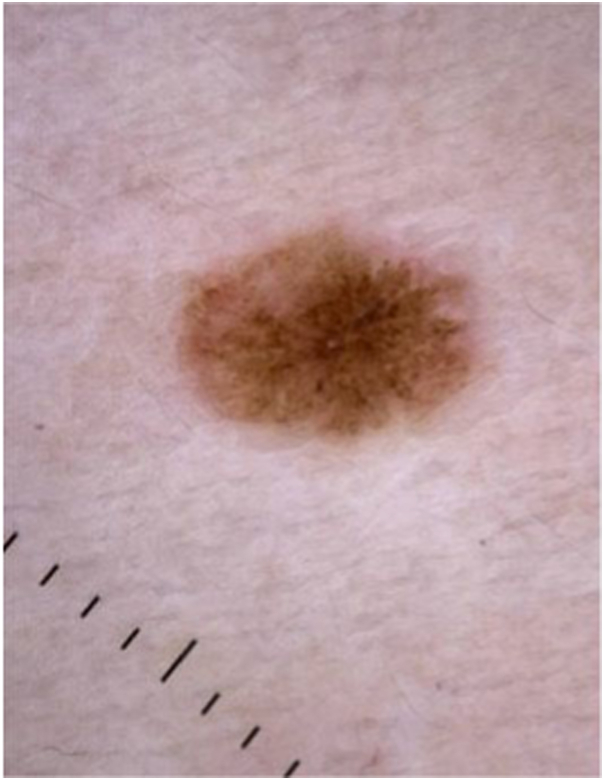
Dermoscopic image showing *starburst* pattern with *light pink* background.

A 25-year-old woman presented to clinic concerned about a mole on her left chest. The patient reported the development of a bleeding blister over the mole 6 weeks prior, though she denied any preceding trauma or biopsy. When evaluated, the lesion had healed clinically, but looked atypical on dermoscopic examination. Dermoscopy revealed an atypical pigment network, irregular streaks and a light pink background (Fig 1). The differential diagnosis included a traumatized nevus, melanoma, or an atypical nevus. The patient was referred for further evaluation with reflectance confocal microscopy (RCM).

### RCM

**Fig 2:**
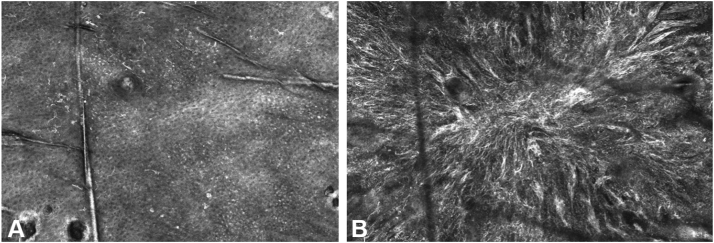
Reflectance confocal microscopy images **(A)** Atypical cells and **(B)** Large nucleotoid cells.

The lesion was imaged using the VivaScope 1500 (Caliber ID) RCM device. Mosaic images were obtained. Confocal imaging revealed numerous large, bright dendritic and round pleomorphic cells in pagetoid spread. An atypical meshwork pattern with irregular junctional thickening was also noted, along with unevenly distributed clusters and single atypical cells within the dermis (Fig 2, *A*, and *B*). These findings were concerning for a melanoma, and a biopsy was performed.

### Histologic diagnosis

**Fig 3:**
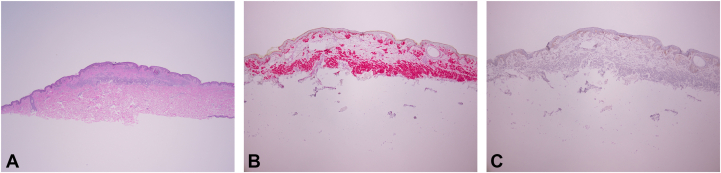
Histopathology of a compound melanocytic nevus with features of recurrent/persistent nevus phenomenon. **A,** Hematoxylin and eosin (H&E, 20× magnification) demonstrates a compound, nested melanocytic proliferation with a zone of superficial dermal fibrosis. The dermal nests demonstrate maturation with depth. **B,** Sox-10 immunostain demonstrates focal crowded growth and low-level upward scatter of single cells limited to the epidermis overlying the fibrosis. **C,** PRAME immunostain is not significantly expressed in the epidermal or dermal melanocytes.

Hematoxylin and eosin-stained sections show a symmetric compound melanocytic proliferation comprising nested melanocytes within the epidermis and dermis, demonstrating maturation with depth (Fig 3, *A*). Centrally, there is a zone of superficial dermal fibrosis, overlying which the epidermal melanocytes are crowded, with occasional low-level upward scatter of single cells that is highlighted by a Sox-10 immunostain (Fig 3, *B*). PRAME is largely negative (Fig 3, *C*).

## Discussion

While dermoscopy is valuable for differentiating benign from malignant melanocytic lesions, certain lesions, such as recurrent nevi (RN), remain diagnostically challenging. RN are benign melanocytic proliferations that regrow after incomplete excision or trauma.^1^ These lesions can exhibit features that overlap with melanoma, making it difficult to distinguish based on clinical and dermoscopic examination alone.^2^ When pigmentation reappears, other than reviewing the original biopsy results, 1 distinguishing feature is that RN typically demonstrate pigmentation confined within the scar, whereas recurrent melanoma may show pigmentation extending beyond the scar. In our case, the absence of a prior biopsy and lack of a scar complicated the assessment. Therefore, RCM referral was essential for management, considering a benign-appearing lesion on RCM could have avoided the need for a biopsy.

RCM has proven to be cost-effective and highly accurate in the diagnosis of melanocytic lesions.^3^ However, features seen in melanoma, such as pagetoid spread or epidermal disarray, can also be seen in other lesions, including spitz nevi or inflamed lesions. A previous study reported that RN on RCM typically lack pagetoid or lateral spread of melanocytes and atypical nests at the junction.^1^^,^^4^ However, this was not the case for our patient. Because the main limitation for RCM is its limited imaging depth, we were able to see the epidermal atypia and superficial fibrosis, but not the benign nevi component in the dermis below the scar.^5^ This begs the question, is RCM an appropriate tool when the differential includes RN versus melanoma? While RCM can decrease the number of unnecessary biopsies, in this setting, it may serve as an extra step that still necessitates a biopsy.

## Conflicts of interest

None disclosed.
